# *Xylella fastidiosa*: Host Range and Advance in Molecular Identification Techniques

**DOI:** 10.3389/fpls.2017.00944

**Published:** 2017-06-08

**Authors:** Paolo Baldi, Nicola La Porta

**Affiliations:** ^1^IASMA Research and Innovation Centre, Fondazione Edmund MachTrento, Italy; ^2^MOUNTFOR Project Centre, European Forest InstituteTrento, Italy

**Keywords:** grape pierce's disease, citrus variegated chlorosis, OQDS, CoDiRO, *Xylella* diagnosis, asymptomatic, leaf scorch disease, quarantine organism

## Abstract

In the never ending struggle against plant pathogenic bacteria, a major goal is the early identification and classification of infecting microorganisms. *Xylella fastidiosa*, a Gram-negative bacterium belonging to the family *Xanthmonadaceae*, is no exception as this pathogen showed a broad range of vectors and host plants, many of which may carry the pathogen for a long time without showing any symptom. Till the last years, most of the diseases caused by *X. fastidiosa* have been reported from North and South America, but recently a widespread infection of olive quick decline syndrome caused by this fastidious pathogen appeared in Apulia (south-eastern Italy), and several cases of *X. fastidiosa* infection have been reported in other European Countries. At least five different subspecies of *X. fastidiosa* have been reported and classified: *fastidiosa, multiplex, pauca, sandyi*, and *tashke*. A sixth subspecies (*morus*) has been recently proposed. Therefore, it is vital to develop fast and reliable methods that allow the pathogen detection during the very early stages of infection, in order to prevent further spreading of this dangerous bacterium. To this purpose, the classical immunological methods such as ELISA and immunofluorescence are not always sensitive enough. However, PCR-based methods exploiting specific primers for the amplification of target regions of genomic DNA have been developed and are becoming a powerful tool for the detection and identification of many species of bacteria. The aim of this review is to illustrate the application of the most commonly used PCR approaches to *X. fastidiosa* study, ranging from classical PCR, to several PCR-based detection methods: random amplified polymorphic DNA (RAPD), quantitative real-time PCR (qRT-PCR), nested-PCR (N-PCR), immunocapture PCR (IC-PCR), short sequence repeats (SSRs, also called VNTR), single nucleotide polymorphisms (SNPs) and multilocus sequence typing (MLST). Amplification and sequence analysis of specific targets is also mentioned. The fast progresses achieved during the last years in the DNA-based classification of this pathogen are described and discussed and specific primers designed for the different methods are listed, in order to provide a concise and useful tool to all the researchers working in the field.

## Introduction

*Xylella fastidiosa* is a Gram-negative, slow growing and strictly aerobic bacterium in the family *Xanthmonadaceae*. It is a widely distributed plant pathogen as it can colonize the xylem of many different species, causing a variety of diseases such as Pierce's disease (PD) in grape (*Vitis vinifera*) or citrus variegated chlorosis (CVC) (Purcell, 2013). *X. fastidiosa* can move upstream and downstream along plant xylem, thanks to the presence of long type IV pili (Li et al., 2007). The bacteria actively multiply until, in the later stages of infection, they block the plant xylem by forming a biofilm. As a consequence, water stress and nutritional deficiencies can occur in the host plant, causing the appearance of disease symptoms (Hopkins, 1989). The first report about a disease caused by *X. fastidiosa* dates back to the end of the nineteenth century, when the so called “California vine disease” destroyed about 14,000 ha of grapes in the Los Angeles area (CA, USA). Newton Pierce (1856–1916), a bacteriologist, was assigned to study the epidemic and even though he was not able to identify the causal agent, he came to the conclusion that the disease was likely caused by a microscopic infectious agent (Pierce, 1892). The disease was named Pierce's disease in 1939, in a bulletin of the California Department of Agriculture (Gardner, 1974), but for a long time the etiological agent was thought to be a virus, until it was recognized as a bacterium in 1973 (Goheen et al., 1973; Hopkins and Mollenhauer, 1973). Pure cultures of the bacterium were isolated from grape in 1978 (Davis et al., 1978) and finally, in 1987, the causal agent of PD was properly classified and named *Xylella fastidiosa* (Wells et al., 1987).

Since the first report in grape, *X. fastidiosa* was isolated and identified from an increasingly large number of plant hosts, with or without symptoms, and recognized to be the causal agent of different diseases(Moller et al., 1974; Hearon et al., 1980; Chang et al., 1993; Grebus et al., 1996; McElrone et al., 1999; Hopkins and Purcell, 2002; Montero-Astua et al., 2008). In many cases also wild plant species were found to carry this pathogen, but often in a latent stage only (Freitag, 1951; Raju et al., 1983; Hopkins and Adlerz, 1988; Blake, 1993; Hill and Purcell, 1997; Li et al., 2001). The distribution range of *X. fastidiosa* is usually limited to tropical and subtropical areas, being its optimal growing temperature 26–28°C (Feil and Purcell, 2001). In some cases strains of *X. fastidiosa* have been found in much colder countries, such as Canada (Goodwin and Zhang, 1997), even if usually this pathogen does not occur in areas with low winter temperatures, such as New York and the Pacific Northwest of USA, or at high altitudes (Hopkins and Purcell, 2002). To date, most of the diseases caused by *X. fastidiosa* have been reported from North and South America. According to the EPPO Global Database (https://gd.eppo.int/taxon/XYLEFA/distribution), only few cases have been reported outside this area, like in Yugoslavia (Berisha et al., 1998), Switzerland (EPPO, 2015a), France (EPPO, 2015b; Marcelletti and Scortichini, 2016b; Denancé et al., 2017), Germany (EPPO, 2016), Iran (Amanifar et al., 2014), and Taiwan (Leu and Su, 1993). In Europe *X. fastidiosa* was first recorded in Puglia region (southern Italy, province of Lecce), where it was recognized to be the causal agent of a dangerous disease of olive trees, the so-called olive quick decline syndrome (OQDS) (Elbeaino et al., 2014; Loconsole et al., 2014).

In the beginning *X. fastidiosa* was regarded as an extended group of bacteria capable of infecting a wide range of host plants and it was only in the early nineties, with the introduction of DNA-based genotyping techniques, that the researchers started to divide the species into different genetic groups (Chen et al., 1992). To date, at least five different subspecies of *X. fastidiosa* have been reported and classified: *X. fastidiosa* subsp. *fastidiosa*, in one report called subsp. *piercei* and then corrected to *fastidiosa* according to the rules (Rule 13d) of the International Code of Nomenclature of Bacteria (Schaad et al., 2004); *X. fastidiosa* subsp. *multiplex*; *X. fastidiosa* subsp. *pauca*; *X. fastidiosa* subsp. *Sandyi*, and *X. fastidiosa* subsp. *tashke* (Schaad et al., 2004; Randall et al., 2009; Janse and Obradovic, 2010). The subsp. *sandyi* is associated with disease in oleander, *Jacaranda* spp., daylily and magnolia (Schuenzel et al., 2005; Hernandez-Martinez et al., 2007). Recently, a new subspecies, *X. fastidiosa* subsp. *morus*, has been proposed (Nunney et al., 2014c). Moreover, it cannot be ruled out that other subspecies still exist, as till now most of the studies about the genetic diversity of *X. fastidiosa* have been performed on cultivated crops of relevant economic importance, while little is known about the strains that colonize wild grasses, sedges and forest trees. Therefore, the development and application of molecular methods to the study of *X. fastidiosa*'s genetic diversity can be of primary importance to fill such gap and extend our knowledge about the true diversity of this organism.

Many sap-feeding insects can function as vectors for the transmission of *X. fastidiosa* to host plants, especially sharpshooters and froghoppers or spittlebugs (*Cicadellidae*) (Janse and Obradovic, 2010; Bhowmick et al., 2016). After acquisition from the source plant, the bacterium is persistent in the vector (Severin, 1949) and can multiply in the foregut (Brlansky et al., 1983; Hill and Purcell, 1995). The process of acquisition and transmission of *X. fastidiosa* by the vector is very complex and can be dependent on many variables such as host plant, vector species and bacterium subspecies in interaction environmental variables, first at all climate. Nevertheless, one important factor has been proven to influence the efficiency of acquisition, that is *X. fastidiosa* population size (number of live cells per gram of plant tissue) (Hill and Purcell, 1997). In fact, the feeding apparatus of vectors is not an easy environment to be colonized, due to the fast flow of sap, that was estimated to reach an average speed of 8 cm/s (Purcell et al., 1979) and the possible turbulence caused by the fast contractions of the muscles allowing the insect to pump sap from the plant, that are contracted and relaxed approximately once every second (Dugravot et al., 2008). Therefore, it is likely that only few cells, out of the thousands acquired by the vector when feeding from an infected plant, actually succeed to colonize the vector's foregut. Another factor that can influence the efficiency of *X. fastidiosa* transmission, and particularly inoculation, is a long access period of the vector to the plant, as probably longer periods allow the insects to deliver a greater number of bacteria, having time to generate a large number of inoculation events (Almeida and Purcell, 2003).

The initial belief that *X. fastidiosa* was a generalist pathogen capable of infecting a very large range of host plants has gradually changed with the discovery and characterization of genetically different strains of *X. fastidiosa*, each capable of infecting distinct hosts. Also the infection characteristics can vary considerably in different species. In some hosts the bacteria multiply locally but cannot move and eventually the infection can regress spontaneously (Purcell and Saunders, 1999). In the past, reciprocal transmission tests have been conducted for *X. fastidiosa* strains. For example, the strains infecting grape cannot infect peach (*Prunus persica*) and peach strains cannot infect grape (Hopkins, 1989). Similarly, grape strains cannot infect oleander (*Nerium oleander*) and *vice-versa* (Purcell et al., 1999), while a CVC strain produced leaf scorch disease in coffee (*Coffea arabica*) (Li et al., 2001) and CVC and coffee strains can infect grape (Hopkins and Purcell, 2002). Natural isolates produced leaf scorch disease in American elm tree but failed in reciprocal transmission in sycamore (Sherald, 1993). However, in this puzzling host/pathogen interaction the host specificity of *X. fastidiosa* is probably a genetically-controlled character, even though in the genome of this bacterium no genes coding for effector proteins were found, nor it is present a type III secretion system (Van Sluys et al., 2003). Pathogenicity factors have been found in *X. fastidiosa* under the control of a cell-cell signaling system, similar but not equal to the one found in the sister genus *Xanthomonas* (Chatterjee et al., 2008). By altering such cell-cell signaling system it is possible to modify *X. fastidiosa* host specificity (Killiny and Almeida, 2011).

## Hosts and diseases

As already mentioned above, *X. fastidiosa* can infect a great number of plant species. A partial list of the main hosts is shown in Table 1 (EFSA, 2016). At present, according to the European Food Safety Authority (EFSA), the updated list of *X. fastidiosa* hosts consists of 359 plant species (including hybrids) from 75 different plant families (EFSA, 2016). Even if the infection process is always the same, the symptoms and the diseases caused by *X. fastidiosa* may vary among species. In addition to PD (Stevenson et al., 2005), CVC and ALS, the most known and dangerous diseases are phony peach disease (PPD) in peach and a number of leaf scorch diseases such as oleander leaf scorch (OLS), coffee leaf scorch (CLS) and plum (*Prunus domestica*) leaf scald (PLS).

**Table 1:** Partial list of the main plant hosts of *Xilella fastidiosa* and their *X. fastidiosa* subspecies.

**Host scientific name**	**Type of infection**	**EPPOCode**	**Subspecies**
*Acacia saligna*	Incidental	ACASA	*pauca*
*Acer rubrum*	Incidental	ACRRB	*multiplex*
*Carya illinoinensis*	Minor	CYAIL	*multiplex*
*Citrofortunella microcarpa*	Minor	CJFMI	nd
*Citroncirus*	Minor	1CJCG	nd
*Citrus*	Minor	1CIDG	*pauca, fastidiosa*
*Citrus sinensis*	**Major**	CIDSI	*pauca*
*Coffea sp*.	**Major**	COFSS	*pauca (BRA)*
*Coffea sp*.	**Major**	COFSS	*fastidiosa (C.Rica)*
Cyperaceae	Wild/Weed	1CYPF	nd
*Fortunella*	Minor	1FOLG	nd
*Liquidambar styraciflua*	Incidental	LIQST	*multiplex*
*Medicago sativa*	Minor	MEDSA	*fastidiosa*
*Morus alba*	Incidental	MORAL	*morus*, (former *multiplex, sandyi)*
*Morus rubra*	Incidental	MORRU	*fastidiosa*
*Nerium oleander*	**Major**	NEROL	*sandyi*
*Olea europaea*	**Major**	OLVEU	*pauca* (ITA, ARG, BRA)
*Olea europaea*	**Major**	OLVEU	*multiplex* (USA, FRA)
*Persea americana*	Incidental	PEBAM	nd
*Platanus occidentalis*	Minor	PLTOC	*multiplex*
*Poaceae*	Wild/Weed	1GRAF	nd
*Polygala myrtifolia*	**Major**	POGMY	*pauca* (ITA)
*Polygala myrtifolia*	**Major**	POGMY	*multiplex* FRA)
*Poncirus trifoliata*	Minor	PMITR	nd
*Prunus angustifolia*	Incidental	PRNAN	nd
*Prunus armeniaca*	Minor	PRNAR	*multiplex*
*Prunus avium*	Minor	PRNAV	*pauca* (ITA)
*Prunus avium*	Minor	PRNAV	*fastidiosa* (USA)
*Prunus cerasifera*	Incidental	PRNCF	*multiplex*
*Prunus domestica*	Minor	PRNDO	*multiplex*
*Prunus dulcis*	Minor	PRNDU	*multiplex-fastidiosa* (USA)
*Prunus dulcis*	Minor	PRNDU	*pauca* (ITA)
*Prunus persica*	**Major**	PRNPS	*multiplex, fastidiosa*
*Prunus salicina*	Minor	PRNSC	*multiplex*
*Quercus palustris*	Minor	QUEPA	*multiplex*
*Quercus rubra*	Minor	QUERU	*multiplex*
*Sorghum halepense*	Wild/Weed	SORHA	nd
*Spartium junceum*	Incidental	SPUJU	*fastidiosa* (USA)
*Spartium junceum*	Incidental	SPUJU	*multiplex* (FRA), *pauca* (ITA)
*Ulmus americana*	Minor	ULMAM	*multiplex*
*Vaccinium corymbosum*	Minor	VACCO	*multiplex*
*Vaccinium virgatum*	Minor	VACVG	nd
*Vinca minor*	Incidental	VINMI	*pauca* (ITA)
*Vitis*	Minor	1VITG	*fastidiosa*
*Vitis labrusca*	Minor	VITLA	*fastidiosa*
*Vitis vinifera*	**Major**	VITVI	*fastidiosa*
*Westringia fruticosa*	Incidental	WESRO	*pauca* (ITA)
*woody plants*	Wild/Weed	2WOOP	*multiplex*

*Source: EPPO Global Database (https://gd.eppo.int/taxon/XYLEFA/hosts), and EFSA Journal database (EFSA, 2016). Major infections are indicated in bold*.

### Grape (PD)

Symptoms of Pierce's disease may vary according to the characteristics of the infected cultivar as well as to the time of infection and seasonal factors (Goheen, 1988). Plants that were infected the previous growing season will generally show more severe symptoms when compared to those infected only in the current season. The first signs of the disease are a sudden drying and yellowing of the leaf margins (a red band can be present in red cultivars), due to the occlusion of leaf veins by bacterial infestation (Hopkins, 1981; Newman et al., 2003). Eventually, the affected (scorched) leaves will fall, usually from the distal part of the petiole, leaving the leaf stems attached to the cane (matchsticks) (Stevenson et al., 2005). Severely infected plants can be completely defoliated within late summer. During the first year of infection, the symptoms are limited to one or few shoots, but get worse over time. The seasonal concentration of bacteria in infected tissues is variable, being at a maximum in late spring and early summer. Usually, *X. fastidiosa* is not detectable in shoots from current season during the first 4 weeks of growth, but it's detectable in old wood (Hopkins, 1981). New wood will mature irregularly, producing patches of brown and green bark (the so called green islands), especially in the intermediate zone of a shoot, between the green tip and the browned basal part (Stevenson et al., 2005). The tips of canes may eventually die back and the new shoots will be shorter and stunted. Also fruit production will be progressively reduced, with most of the fruit clusters drying and wilting. In 1–5 years, depending on the susceptibility of the different genotypes, the infected plants usually die, even if differences may be due also to the climate and particularly to water stress (Thorne et al., 2006). Indeed, for many years most of the symptoms of PD were attributed to the limited water transport due to vessels occlusion (Hopkins, 1989) but recently it was shown that plants infected with *X. fastidiosa* show symptoms that are characteristic of PD and cannot be reproduced by water deficit (Thorne et al., 2006).

### Citrus (CVC)

*X. fastidiosa* can infect any type of citrus species and hybrids but the severity of symptoms may vary according to the genotype of the host. Sweet oranges are the most susceptible, while grapefruit, mandarins, lemons, limes and trifoliate orange are only moderately susceptible (Garcia et al., 2012; Gmitter et al., 2012; Casais et al., 2014; Fadel et al., 2014). In most cases CVC is not lethal but infected trees always display a reduced vigor and growth rate and a decreased productive lifespan. Symptoms develop faster in young trees, that are also more susceptible to new infections (Garcia et al., 2012) Symptoms can be easily confused with zinc deficiency, as plants show chlorotic spots on the upper surface of leaves, especially in the interveinal area (Beretta et al., 1997). Deficiencies of P and K have been reported in leaves of CVC affected trees, together with high concentrations of Fe, Mn, and Zn (Silva-Stenico et al., 2009). On the leaves, chlorotic lesions appear on the upper side, while on the lower leaf side gummy lesions may appear, due to the production by *X. fastidiosa* of fastidian gum, an exopolysaccharide that was proposed to be involved in the formation of biofilms that allow the attachment and survival of bacteria inside the xylem vessels (da Silva et al., 2001). While the leaf matures, the gummy lesions can enlarge and become necrotic. Fruits are also affected and this represents one of the major problems of CVC, especially from an economical point of view. Affected fruits remain smaller (thinning does not occur on infected branches), become hard and ripen earlier. The color is the same as healthy fruits but the juice content is reduced, while acidity is higher (Gonçalves et al., 2014). Bacteria can be found also in roots of infected plants (Hopkins et al., 1991).

### Peach (PPD)

Symptoms of PPD are not immediately apparent on host plants until 1 year or more from the first infection. Infected plants usually show a reduced internode length of new growth, giving to the tree a rather bushy aspect (Hutchins, 1933). Leaves become flattened and dark-green. In spring, trees infected by *X. fastidiosa* flower and leaf earlier than normal and hold their foliage longer in the fall (Wells et al., 1981; Hopkins and Purcell, 2002). Unlike other diseases caused by *X. fastidiosa*, PPD is not lethal to the infected plants. Nevertheless, PPD may cause significant damage to orchards as one of the main consequences of the infection is loss of fruit production. If trees become infected prior to production age, they will never produce fruits, while plants infected after production age will exhibit reduced production and smaller and highly colored fruits that are not suitable for the market (Evert, 1985). After 2–4 years after first infection, diseased trees may stop producing fruit at all. In peach, *X. fastidiosa* was detected in roots xylem fluid both in symptomatic or asymptomatic trees (Aldrich et al., 1992).

Unlike other *Prunus* species such as almond and plum, peach does not show leaf scorch symptoms when infected by *X. fastidiosa* (Ledbetter and Rogers, 2009).

### Other hosts (scorch diseases)

Many plant species, when infected by *X. fastidiosa*, show very similar symptoms, especially on the leaves, and namely: early after infection a slight chlorosis appears, usually along the margins of leaves. In some species (e.g., coffee, olive), these symptoms may affect initially younger shoots, while in others (e.g., elm) may progress from older to younger leaves (Sherald, 1993) and this may confound the early diagnosis. Symptoms on leaf petioles of infected plants colonized by *X. fastidiosa* varied from coffee, plum, and sweet orange in clear decreasing order of severity (Alves et al., 2004). In all cases symptoms get worse with time, even if differences may exist depending on the genetic background of the host plant, timing of inoculation and overwinter survival of the pathogen (Cao et al., 2011). The disease gradually extend from one or few branches to the entire crown and the leaves may desiccate completely and eventually fall. One of the major problems caused by *X. fastidiosa* infections is the decreased fruit quality and yield in commercially important crops such as coffee and olive (Rocha et al., 2010; Della Coletta et al., 2016) Plants affected by scorch disease usually show weak and stunt growth, may be more susceptible to environmental stresses, such as water or heat stress, but do not always die. Instead, they can be removed to reduce the risk of new infections or because the weakened plant has become dangerous (e.g., ornamental trees). The problem of *X. fastidiosa* infection is getting particularly serious in the south-eastern part of Italy on olive trees as a strain of *X. fastidiosa* subsp. *pauca* was strongly associated to a severe burst of OQDS (Saponari et al., 2013). Only few years ago (2008–2010) the first cases where reported. Nowadays, according to the last surveys, an area of at least 10,000 ha in the province of Lecce (Salento) could be infected by *X. fastidiosa* (Martelli et al., 2016). Only recently two olive cultivars, Leccino and Favolosa FS-17 were selected, that appear to have some degree of tolerance to the disease (Giampetruzzi et al., 2016). The introduction in Puglia of *X. fastidiosa* could be due to infected plant material imported from Central America (Giampetruzzi et al., 2015a; Marcelletti and Scortichini, 2016b). Other recent reports describe the presence of *X. fastidiosa* in olive trees showing leaf scorch symptoms in Argentina (Haelterman et al., 2015) and Brazil (Della Coletta et al., 2016). The two South American strains resulted different from each other and from the Italian strain but were both classified as belonging to the subsp. *pauca*. Over the last few years, the presence of *X. fastidiosa* outside the American continent is becoming more and more common. In October 2015, the bacterium (*X. fastidiosa* subsp. *multiplex*) was discovered in France, initially on the island of Corsica, on *Polygala myrtifolia* plants (ornamentals) and later on the mainland (*X. fastidiosa* subsp. *multiplex*) (EPPO, 2015b), with almost 300 foci found and nearly 30 host plant species declared contaminated (Denancé et al., 2017). In order to prevent entry and spread of *X. fastidiosa* within the European Union territory, specific EU phytosanitary measures have been taken (PM 7/24 (2) 2016. *Xylella fastidiosa*. EPPO Bull, 46: 463–500. doi: 10.1111/epp.12327).

### Detection and identification

Detection and identification of *X. fastidiosa* is not an easy task. First of all, these bacteria are slow-growing (up to 2–3 weeks can be necessary to obtain colonies on agar media) and secondly many common culture media are not suitable to grow *X. fastidiosa* strains. Instead, selective media (PD2, PW, CS20) should be used (Schaad et al., 2001). Direct identification of bacteria is possible only using dark field or phase contrast microscopy, due to the dimensions of *X. fastidiosa* cells (0.2–0.4 μm radius and 0.9–3.5 μm length) (Wells et al., 1987). In addition, microscopy identification requires a quite specific professional training to be efficient. Immunological methods, such as enzyme-linked immunosorbent assay (ELISA) can be used (Chang et al., 1993; Leu et al., 1998). More recently, detection of *X. fastidiosa* by immunofluorescence technology has been described (Carbajal et al., 2004; Buzkan et al., 2005). Anyway, most of the antibody-based detection assay are effective only at the species level, while at our knowledge a single report is available describing the development of single chain variable fragment (scFv) antibodies specific for *X. fastidiosa* subsp. *pauca* (Yuan et al., 2015). Another detection method that since the nineties has become more and more popular relies on PCR amplification of bacterial DNA. Several protocols have been developed employing different techniques, all with the use of specific primers capable of recognizing target sequences on genomic DNA. The PCR-based detection methods are usually faster and cheaper than standard plating methods and more specific than immunological methods as it is often possible to design primers specific for the desired genus, species or sub-species. Despite all these advantages, some drawbacks are still present in the use of DNA amplification methods. First of all specific equipment is required and often the protocols must be optimized in order to work properly with the specific samples and conditions of a given laboratory. Then, even when all the technical requirement are met, there is still the possibility to detect false-positive or false-negative for several reasons. For example, the PCR-based methods all allow detection of bacterial DNA, that in some cases can still persist in the environment even when the bacterial cells are no longer viable (Willerslev and Cooper, 2005). Therefore these methods alone cannot be used to distinguish between viable and non-viable bacteria, with exception of few cases (Gedalanga and Olson, 2009). The genetic material for PCR amplification can be isolated with standard protocols, which may be optimized according to the user's needs, or using several commercial kits, many of which are suitable for robotized DNA extraction and allow the simultaneous processing of up 96 samples (Smit et al., 2001). In order to avoid the laborious and time-consuming step of DNA purification, several attempts were made using directly plant sap as a template, not always with reliable results (Minsavage et al., 1994; Banks et al., 1999).

The DNA-based methods are rapidly becoming the most widely used in all modern laboratories for the detection and identification of *X. fastidiosa* strains infecting a number of plant species and new protocols and primers are continuously being developed. Here follows a review of the most commonly used PCR approaches (summarized in Table 2).

**Table 2:** Most commonly used PCR-based techniques for *X. fastidiosa* identification.

**Molecular method**	**Advantages**	**Disadvantages**	**References**
Classic PCR	High sensitivity, specificity and accurate results for the detection of *Xylella* and its subspecies also in non-axenic conditions. Many applications in molecular analysis. Easy diagnostic interpretation	Unable to quantify the target DNA, only qualitative test. Some metabolites or contaminants in the sample can interfere with PCR performance. PCR conditions must be optimized in each host and environment for better performance	Firrao and Bazzi, 1994; Minsavage et al., 1994; Pooler and Hartung, 1995a; Banks et al., 1999; Rodrigues et al., 2003; Huang and Sherald, 2004; Chen et al., 2005; Travensolo et al., 2005; Hernandez-Martinez et al., 2006; Martinati et al., 2007; Morano et al., 2008; Huang, 2009; Livingston et al., 2010; Melanson et al., 2012; Guan et al., 2015; Bleve et al., 2016
RAPD	Useful to study unknown species where there is not *apriori*. knowledge of sequencing. Quite useful to detect high polymorphisms. Limited cost, Simple and rapid. Small amount of template DNA required. The amplification products can be further characterized	Markers are dominant. Reproducibility can be low among labs and with different polymerases and facilities, especially when not using random primers with high annealing temperature. In many cases standardization of the protocol for each lab is required. Nowadays this technique is considered obsolete	Denny et al., 1988; Hartung and Civerolo, 1989; Grajalmartin et al., 1993; Chen et al., 1995, 2002; Pooler and Hartung, 1995a; Albibi et al., 1998; Rosato et al., 1998; Banks et al., 1999; Hendson et al., 2001; Lacava et al., 2001; Qin et al., 2001; Su et al., 2008
RFLP	It was one of the first methods used for genetic fingerprinting, The basic RFLP analysis is no longer used. Variations exist such as terminal restriction fragment length polymorphism (TRFLP), which may still have applications related to the characterization of bacteria. By PCR-RFLP, the hybridization step can be skipped	Obsolete technique. Relatively large amount of DNA is required. RFLP approach is tedious and requires numerous steps that may take weeks to yield results. Relatively high cost and low polymorphism	Chen et al., 1992; Rosato et al., 1998; Mehta et al., 2001; Qin et al., 2001; Picchi et al., 2006
qRT-PCR	It allows not only the identification, but also the quantification of bacteria in real time. High sensitivity, specificity and reproducibility. Relatively fast method. It is possible to use also variations in melting temperature to differentiate strains of bacteria	Expensive equipment and reagents are required. Setting up and optimization of the protocol require specific technical skills as well the interpretation of results	Oliveira et al., 2002; Schaad et al., 2002; Bextine et al., 2005; Francis et al., 2006; Bextine and Child, 2007; Choi et al., 2010; Harper et al., 2010; Brady et al., 2012; Guan et al., 2013; Li et al., 2013; Ionescu et al., 2016
SSR	Simple lab procedure, relatively low costs to start, based on PCR termocycler. High level of polymorphism and relatively low amount of target DNA required. Co-dominant markers. The reproducibility is quite good	Previous knowledge of the genomic sequence is required to design specific primers, thus SSRs are limited primarily to economically important species. Point mutations at the site of primer annealing could lead to occurrence of null alleles	Della Coletta-Filho et al., 2001; Coletta and Machado, 2003; Lin et al., 2005, 2013, 2015; Montero-Astua et al., 2007; Montes-Borrego et al., 2015; Francisco et al., 2017
MLST	Highly discriminatory nucleotide sequence based method of characterization based on the sequencing of approximately 450-bp internal fragments of seven housekeeping genes amplified by PCR. This approach is particularly helpful for the typing of bacterial pathogens. The system is very sensitive to discriminate *X. fastidiosa* subspecies and strains in rapid real time reactions. The major advantage of MLST is the possibility to compare the results obtained in different studies. It may also be used to address basic questions about evolutionary and population biology of bacterial spp.	The analysis of only seven loci may limit the sensitivity, especially when close strains are analyzed. Sequencing of the PCR products using an automated sequencer is required. For that, MLST is not always suitable for routine infection controls or outbreak investigation due to relatively high cost and lack of broad access to high-throughput DNA sequencing	Scally et al., 2005; Schuenzel et al., 2005; Almeida et al., 2008; Yuan et al., 2010; Brady et al., 2012; Nunney et al., 2012, 2013, 2014a,b,c; Parker et al., 2012; Elbeaino et al., 2014; Harris and Balci, 2015; Marcelletti and Scortichini, 2016a; Bergsma-Vlami et al., 2017; Coletta-Filho et al., 2017; Denancé et al., 2017; Kandel et al., 2017
Multiplex PCR	Costs are reduced when compared to standard PCR as well as reaction volumes. It allows rapid detection also of multiple strains simultaneously. Close tube system limits the risk of contamination	Primer design is the critical point, they can interfere each other giving false negative (genes or bacteria undetected). Skilled personnel is required to perform the test	Rodrigues et al., 2003; Choi et al., 2010; Myers et al., 2010; Lopes et al., 2014; Jacques et al., 2016
Nested PCR	Improved sensitivity and specificity when compared with classical PCR methodology. Useful technique for studying molecular epidemiology in the field	The protocol may be a little more difficult to optimize than for standard PCR. More time consuming and expensive than normal PCR. Unable to quantify the target DNA	Pooler et al., 1997; Buzkan et al., 2003; Ciapina et al., 2004; Huang, 2007; Silva et al., 2007; Lopes et al., 2014

### PCR detection with specific primers

Since the first half of the nineties specific PCR primers have been used in order to identify *X. fastidiosa* from infected plants. In the first reports, the genomic region amplified could belong to a defined gene, such as 16S rDNA (Firrao and Bazzi, 1994), but also to selected fragments of bacterial DNA of unknown function (Minsavage et al., 1994). In Pooler and Hartung (1995a) developed a series of specific primers capable to detect *X. fastidiosa* in general and *X. fastidiosa* strains that cause CVC by cloning and sequencing randomly amplified polymorphic DNA (RAPD) products (Pooler and Hartung, 1995a). Since then a number of reports have been published describing the development of specific PCR primers for the identification of *X. fastidiosa* (Table 3) and if the 16S rDNA and the 16S-23S intergenic spacer region (ITS) are the most common targets (Rodrigues et al., 2003; Chen et al., 2005; Martinati et al., 2007), also several other genomic sequences, with or without a known function, have been used (Travensolo et al., 2005; Huang, 2009). The use of different genes, alone or in combinations, can be exploited in order to increase the level of sensitivity and specificity of the test. As a matter of fact, the early detection of pathogens before the development of any symptom by the plant host is crucial for the development of a correct defense strategy but when used for field analysis the detection test must be also as easy and fast as possible. Therefore, it is difficult to develop a single test that can be used in all conditions, but combining different approaches the researchers can find a balance among sensitivity, specificity and ease of use. As an example, the gene encoding the b-subunit polypeptide of the DNA gyrase (*gyrB*) was used in combination with the 16S rDNA in order to increase the specificity of the test, because *gyrB* is thought to evolve much faster than 16S rDNA (Yamamoto et al., 1999), therefore allowing a higher resolution when comparing closely related strains of bacteria (Rodrigues et al., 2003). For an initial genetic analysis of the population of *X. fastidiosa* in Texas, *gyrB* was used together with *mopB*, the latter coding for an outer membrane protein of the OmpA family with a fairly conserved sequence (Morano et al., 2008). When using a single primer pair to amplify a specific target, there is a low but non-zero possibility of false-positives due to primer recognition of non-target DNA with high sequence similarity to the target. On the contrary, false-negatives can be generated due to variations in bacterial genome, particularly to mutations in the recognition site of the primers (Scally et al., 2005). To avoid such problems, more complex approaches can be used. In a recent study, *gyrB* was combined with a set of housekeeping genes for the development of an array-PCR protocol that allowed to analyze a large number of samples avoiding the occurrence of false negatives due to the failure of PCR amplification of a single primer (Livingston et al., 2010).

**Table 3:** List of specific primers designed for the PCR-based identification of *X. fastidiosa*.

**Forward primer**	**Reverse primer**	**Size**	**Target**	**Hosts**	**Citations WoS - G-Scholar**	**References**
**RST31:** GCGTTAATTTTCGAAGTGATTCGA	**RST33:** CACCATTCGTATCCCGGTG	733	7.4 kb EcoR1 restriction fragment	Grapevine, citrus, oak, red oak, sycamore, plum, goldenrod	193–285	Minsavage et al., 1994
**XF1-F:** CAGCACATTGGTAGTAATAC	**XF6_R:** ACTAGGTATTAACCAATTGC	400	16s rDNA	Grapevine, almond, plum, American elm, citrus	(nd 2004) – 16	Firrao and Bazzi, 1994
**208-1:** ACGGCCGACCATTACTGCTG	**208-2:** ACGGCCGACCCGGAGTATCA	320	RAPD fragment	Citrus, grapevine, mulberry, almond, plum, elm, oak, ragweed, periwinkle	119–180	Pooler and Hartung, 1995a
**203-1:** CACGGCGAGTATCGGCTTTC	**203-2:** CACGGCGAGTCGACGTAAAT	2,000	–	–		
**204-1:** TTCGGGCCGTCAATGTGTTG	**204-2:** TTCGGGCCGTTTACTCAAAG	800	–	–		
**230-1:** CGTCGCCCATCAACGCCAAA	**230-2:** CGTCGCCCATTGTGCTGCAG	700	–	–		
**272-1:** AGCGGGCCAATATTCAATTGC	**272-2:** AGCGGGCCAAAACGATGCGTG	700	–	–		
**272-1-int:** CTGCACTTACCCAATGCATCG	**272-2-int:** GCCGCTTCGGAGAGCATTCCT	500	–	–		
**CVC-1:** AGATGAAAACAATCATCGAAA	**272-2:** AGCGGGCCAAAACGATGCGTG	600	–	Citrus		
**CVC-1:** AGATGAAAACAATCATCGAAA	**272-2-int:** GCCGCTTCGGAGAGCATTCCT	500	–	Citrus (specific)		
**XF176f:** AAACAATCACAGGGGACTGC	**XF954r:** CTATCCGAACACTTCCTATG	779	PD-specific RAPD fragment (PD1-1-2)	Grapevine (Pierce's disease)	20–29	Banks et al., 1999
**XF176f:** AAACAATCACAGGGGACTGC	**XF686r:** ATATTCATAGATTCCGTCGA	511	–	Citrus, mulberry, oak, periwinkle, peach, plum (Non-Pierce's disease)		
**0067-a-S-19:** CGGCAGCACATTGGTAGTA	**1439-a-A-19:** CTCCTCGCGGTTAAGCTAC	1,348	16S rDNA	Citrus, grapevine, mulberry	43–70	Rodrigues et al., 2003
**0067-a-S-19:** CGGCAGCACATTGGTAGTA	**0838-a-A-21:** CGATACTGAGTGCCAATTTGC	745	–	–		
**0838-a-S-21:** GCAAATTGGCACTCAGTATCG	**1439-a-A-19:** CTCCTCGCGGTTAAGCTAC	603	–	–		
**FXYgyr499:** CAGTTAGGGGTGTCAGCG	**RXYgyr907:** CTCAATGTAATTACCCAAGGT	429	b-subunit polypeptide of the DNA gyrase (gyrB)	–		
**G1:** GAAGTCGTAACAAGG	**L1:** CAAGGCATCCACCGT	522	16S rDNA	Porcelain berry, wild grape, Mulberry		Huang and Sherald, 2004
**Teme150fc:** TCTACCTTATCGTGGGGGAC	**Teme454rg:** ACAACTAGGTATTAACCAATTGCC	348	16S rDNA	Almond	54–78	Chen et al., 2005
**Teme150fc:** TCTACCTTATCGTGGGGGAC	**Xf16s1031r:** AAGGCACCAATCCATCTCTG	700	–	–		
**Dixon454fa:** CCTTTTGTTGGGGAAGAAAA	**Dixon1261rg:** TAGCTCACCCTCGCGAGATC	847	–	–		
**REP1-R:** IIIICGICGIATCCIGGC	**Xf-1:** CGGGGGTGTAGGAGGGGTTGT	350	Amplified genomic fragment	Citrus, grape, almond, mulberry, oak, periwrinkle, plum, coffee, elm, ragweed	3–7	Travensolo et al., 2005
**XF1968-L:** GGAGGTTTACCGAAGACAGAT	**XF1968-R:** ATCCACAGTAAAACCACATGC	638	Gene XF1968	Almond, oleander	21–42	Hernandez-Martinez et al., 2006
**XF2542-L:**TTGATCGAGCTGATGATCG	**XF2542-R:** CAGTACAGCCTGCTGGAGTTA	412	Gene XF2542	Grape, almond, Spanish broom, *Brassica* spp.		
**ALM1:** CTGCAGAAATTGGAAACTTCAG	**ALM2:** GCCACACGTGATCTATGAA	521	Gene ALM1	Almond (specific)		
**16S-23SF:** GATGACTGGGGTGAAGTCGT	**16S-23SR:** GACACTTTTCGCAGGCTACC	650	16S-23S intergenic spacer	Citrus, coffee, grapevine, mulberry, almond elm, ragweed, periwinkle	1–2	Martinati et al., 2007
**307 BBF:** GCAAGTCAGGGTAGCGTCTC	**943 BBR:** GGCTTCTCTGTCGATTTTCG	nd	mopB	Grape, Sea myrtle, Redspike Mexican hat, others	5–10	Morano et al., 2008
**HQ-OLS08:** TGTACGTCCTGAAACCATCTTG	**HQ-OLS05:** TTCTGGAAGCTTTGAGTAAGGG	274	RAPD fragment	Oleander (specific)	7–10	Huang, 2009
**D056L:** AACAAGGGACCTTCCATGC	**D858R:** AGCAATCGCTGCACCTAAAT	842	Succinyl-CoA synthetase alpha subunit (sucD)	Almond	0–0	Livingston et al., 2010
**G151f:** GATCCGGAAAGTGGGGAGATTACTATC	**G368r:** GCCATTGCAAAAGCAGTACGCTCA	217	DNA polymerase III subunit beta (dnaN)	–		
**A151f:** GATGCGGAAAGCGGGGAGATTACTATT	**A518r:** GCCTTTACGCGGCAAAATAATCTGA	367	DNA polymerase III subunit beta (dnaN)	–		
**A97f:** CATGCTGGTGGTAAGTTCGACGATAAC	**A476r:** CAACAATGCCGTTGTGCTCACCG	379	b-subunit polypeptide of the DNA gyrase (gyrB)	–		
**G223f:** CGGTGGCGAAACGGTAATCC	**G705r:** GGAGAAATGTTTGGCAAAGACAGGC	482	mdh Malate dehydrogenase	–		
**A345f:** TTTTTCAATGTTGGCGACAGGCTTACT	**A705r:** GGAGAAATGTTTGGCAAAGACAGGT	360	mdh Malate dehydrogenase	–		
**B001L:** TTAGGTGGCAAGGATCGAAT	**B462R:** GGGCCGATCAAAATCAATCT	491	ppiB Peptidyl-prolyl cis-trans isomerase	–		
**A284f:** GACGAGTTTGCCAAGTTTGATGATGAAATC	**A680r:** GCCAGTCGAACCCACCAAG	396	gltA Citrate synthase	–		
**I068L:** CGTGGGTCACGAGTCATAAA	**I386R:** TCACACAAAACTACGGCACTG	358	rpsI 30S ribosomal protein S9	–		
**PspB-256f:** TGAGTGCCTGCGGTGGTA	**PspB-256r:** CGAAACTTGGCAGCTAACG	256	pspB Serine protease	–		
**XFPglA_Fw:** GCCTCCGGTGCGACTGCTTC	**XFPglA_Rv:** GCTGCGATTGGACACACATTG	nd	PglA	Pecan, Grapevine, Oleander, sycamore	10–14	Melanson et al., 2012
**Mul-15040-F:** ATTTTCGCGATTTTGGAGTT	**Mul-15040-R:** TTCTTGTGTACTCCGCCTCA	312	Hypothetical protein with putative bacillithiol system oxidoreductase, YpdA family	Mulberry and olive (specific)	2–2	Guan et al., 2015
**Xfa-rpod-F4:** ACTGAGGTTGTCGTTGGCTT	**Xfa-rpod-R4:** CCTCAGGCATGTCCATTTCC	988	RNA polymerase sigma-70 factor (rpoD)	Olive, citrus, coffee	1–2	Bleve et al., 2016
**Xfa-dnaA-2F:** TTCCATCAAATTGACGCGCT	**Xfa-dnaA-2R:** CGGCAAGCATGTAACACTGT	650	Chromosomal replication initiator protein DnaA (dnaA)	–		

Based on the target genomic region or selected bacterial gene, the specificity of the PCR primers used for the analysis can vary, from genus to sub-species and in some cases even to different strains belonging to the same sub-species, so it is crucial a correct primer choice in order to avoid misinterpretation of results. For *X. fastidiosa* analysis, a number of explicative examples can be found in literature. Starting from a pool of *X. fastidiosa*-specific RAPDs, Pooler and Hartung (Pooler and Hartung, 1995a) analyzed a group of 21 bacterial strains collected in different regions of USA and Brazil and developed a pair of primers capable to distinguish *X. fastidiosa* strains causing CVC from all the others. Similarly, Banks and colleagues designed two specific sets of primers from a PD strain collected in Florida: the first could be used to distinguish between *X. fastidiosa* and *Xanthomonas campestris* and the second to amplify a 511 bp fragment from 98 PD strains but not from CVC strains of *X. fastidiosa* (Banks et al., 1999). Therefore, both sets of primers can be exploited to identify *X. fastidiosa* but the second showed a greater specificity and can be used in more detailed studies. More recently a set of primers specific for OLS strains of *X. fastidiosa* was developed and tested successfully on cultured bacteria, infected plant samples and insect vectors capable of transmitting OLS (Huang, 2009). A particular case was reported when a set of primers specific for mulberry-infecting strains of *X. fastidiosa* was developed starting from the nucleotide sequence of a unique open reading frame identified only in mulberry-infecting strains among all the North and South American strains of *X. fastidiosa* sequenced at the date of that work (Guan et al., 2015). Such primers could distinguish between mulberry-infecting strains and those infecting other species, such as sycamore, elm, oak, plum, maple, and grape. Surprisingly, with the same set of primer a specific amplification could be obtained also from two isolates of *X. fastidiosa* belonging the recently sequenced CoDiRO strain infecting olive trees in Italy (Guan et al., 2015). This means that sometimes it can be difficult to identify unambiguously a given strain of *X. fastidiosa* by the use of a single approach. Even the use of advanced techniques such as multilocus sequence typing (described in more details in one of the next sections) is not always enough to distinguish among closely related strains, as for the characterization of a new *X. fastidiosa* strain (Salento-1) infecting olive trees in Italy (Bleve et al., 2016). In this case, the analysis of two additional genes, the polymerase sigma 70 factor (*rpoD*) and the chromosomal replication initiator protein DnaA (*dnaA*) was necessary to separate Salento-1 from closely related strains of *X. fastidiosa* subsp. *pauca* isolated from citrus and coffee in Brazil (Bleve et al., 2016).

A multiprimer combination with three different targets was developed in order to differentiate strains of *X. fastidiosa* infecting grape, almonds and oleander (Hernandez-Martinez et al., 2006). In this way, strains belonging to different subspecies (*multiplex, fastidiosa*, and *sandyi*) could be distinguished. Moreover, two different strains (ALSI and ALSII) belonging to subspecies *multiplex* and infecting almonds could be separated on the basis of amplification patterns. When the aim of the study is to characterize a new strain or a group of strains of *X. fastidiosa* rather than the fast and efficient identification of the pathogen under field conditions, the combination of multiple molecular techniques can greatly enhance the resolution power of the test. A good example is a study performed on pecan, where a multiprimer PCR assay was used in combination with other PCR-based techniques, such as amplification and sequence analysis of the 16S-23S ITS and *pglA* gene as well as enterobacterial repetitive intergenic consensus (ERIC)-PCR and repetitive extragenic palindromic (REP)-PCR. In this way, the authors were able to classify the *X. fastidiosa* strains infecting pecan as belonging to the subsp. *multiplex* (Melanson et al., 2012).

A different way to increase PCR sensitivity is the so-called nested-PCR (N-PCR) that involves the use of two different sets of primers specific for the same region in two successive runs. The second set of primers is designed to recognize a secondary target within the PCR product obtained with the first set. In Pooler et al. (1997) used N-PCR in combination with immunomagnetic separation to screen a population of 16 different species of leafhoppers, putative *X. fastidiosa* vectors, living on American elm (*Ulmus americana* L.) (Pooler et al., 1997). Two of these species regularly tested positive with this technique, with a sensitivity of as few as five bacteria per sample. Similarly, N-PCR was used in combination with immunocapture (IC) for detection of *X. fastidiosa* in grapevine tissue (Buzkan et al., 2003). When compared with standard non-IC-PCR, a 10,000-fold increase of sensitivity was obtained thanks to the IC procedure and a further 1,000-fold increase with N-PCR primers, achieving a maximum sensitivity of 2 cfu/ml of bacteria concentration in grape leaf extract. In another work, three different bacterial extraction and purification protocols were combined with N-PCR and use to identify alternative hosts of *X. fastidiosa* in the Washington D.C. area (McElrone et al., 1999). By the optimization of bacterial DNA extraction method using ionic exchange resin (Chelex 100) and N-PCR, it was possible to increase *X. fastidiosa* detection sensitivity from citrus plants and sharpshooter leafhoppers up to two bacteria per reaction (Ciapina et al., 2004). Therefore, once that primer pairs and PCR conditions have been optimized, N-PCR can be considered a very efficient way to increase detection sensitivity and could be used for the early detection of *X. fastidiosa*.

### Real-time PCR

A further improvement in PCR-based techniques of bacteria detection can be obtained by the use of quantitative real-time PCR (qRT-PCR), that allows not only the identification of the pathogen but also its quantification (Heid et al., 1996; Ionescu et al., 2016). Therefore, by qRT-PCR it is possible to study in more details the temporal and spatial distribution of *X. fastidiosa* in infected plants. The primers used for qRT-PCR are similar to those used for conventional PCR, while the amplification product is usually shorter that in normal PCR. Moreover, depending on the technique used (SYBR green or TaqMan), a fluorescent-labeled probe can be necessary (Heid et al., 1996; Wittwer et al., 1997) (see Supplementary Materials for a list of primers and probes used for *X. fastidiosa* detection). In one of the first reports published, qRT-PCR was used to quantify *X. fastidiosa* in naturally and artificially infected citrus (Oliveira et al., 2002). Temporal differences in bacterial cell number were detected, increasing with the age of the examined leaves. Spatial differences were also found, with no bacteria detected in the upper midrib section of young leaves. In the same work qRT-PCR was used to compare a resistant and a susceptible citrus cultivar. This is a good example of how qRT-PCR can be used to study the development of bacterial diseases in plants. By qRT-PCR it is possible for example to quantify bacteria in different plant organs at different time points after infection, correlate a given amount of bacteria to the appearance of the first symptoms and find differences between plants showing variable degrees of resistance. Nevertheless, qRT-PCR can be used also for field applications. A portable Smart Cycler for 1-h on-site diagnosis was used to detect *X. fastidiosa* in grape (Schaad et al., 2002). Using sap and samples of macerated chips of secondary xylem from trunks of grape trees in a direct qRT-PCR without extraction of DNA, the authors were able to positively detect *X. fastidiosa* in about 26% of the examined asymptomatic plants. The results were then confirmed by other techniques, such as direct isolation of bacteria. Even if these results suggest that qRT-PCR can be used effectively instead of standard PCR, it is always up to the researcher to choose the more suitable approach for each study.

As for conventional PCR methods, also for qRT-PCR the level of specificity can vary according to the genomic target and primer design. As an example, primers HL5 and HL6 (Table S1) were designed to amplify a unique region common to the sequenced genomes of four *X. fastidiosa* strains causing PD, ALS, OLS, and CVC. Such primers can be effectively used to distinguish between infections of *X. fastidiosa* and other plant pathogens and have been tested in several plant and insect species (Francis et al., 2006). In other works, primer sets were specifically developed in order to recognize oleander-infecting strains (Guan et al., 2013) or strains causing CVC (Li et al., 2013). In all, qRT-PCR showed the same advantages as conventional PCR, that are high sensitivity and specificity. Moreover, additional information about temporal and spatial distribution of the pathogen can be obtained. One of the drawbacks of this technique is that it can be more difficult to optimize the protocol, especially when using TaqMan probes. When using qRT-PCR, additional strategies can be used to distinguish among different genotypes. A protocol was developed, based on SYBR green qRT-PCT, for *X. fastidiosa* genotype differentiation using a single primer pair and exploiting differences in melting temperature (T_m_) due to small differences of target sequence. Such protocol was tested on eight PD, six OLS, and six ALS strains that could be successfully placed into the respective strain group by the analysis of T_m_ curve (Bextine and Child, 2007). Similarly but using a set of fluorescent probes, Brady et al. developed a multilocus melt typing (MLMT) system to discriminate *X. fastidiosa* subspecies and strains in rapid real time reactions (Brady et al., 2012). These approaches are potentially very interesting, as theoretically it is possible to detect genetic variations determined by as few as a single base pair alteration. For practical uses, this high sensitivity could be a problem as small unspecific variations in T_m_ curve may lead to misinterpretation of the results. Therefore, a very reliable and reproducible protocol is necessary.

In order to improve sensitivity, speed and ease of detection, several approaches have been tested, such as novel DNA extraction protocols (Bextine et al., 2005; Harper et al., 2010; Yaseen et al., 2015), development of an agar absorption-based technique for elimination of PCR inhibitors (Fatmi et al., 2005) and use of multiplex PCR (Choi et al., 2010; Myers et al., 2010). A novel technique, the loop-mediated isothermal amplification (LAMP), was used as a promising alternative to PCR for *X. fastidiosa* detection as it is easy to use and provides a good reliability and specificity (Notomi et al., 2000). Moreover the LAMP reaction occurs at isothermal conditions, so it can be performed using a simple heat block and does not require any specific equipment. The results are displayed by colorimetric or fluorescent dyes, so the gel running phase of standard PCR is also skipped. The LAMP technique was successfully used to study 20 isolates of *X. fastidiosa* representing the four main subrgroups of the pathogen (Harper et al., 2010) and more recently applied to olive tree and other host plants and insect vectors (Yaseen et al., 2015). However, the main drawback of LAMP when compared with qRT-PCR is a lower sensitivity as the detection limit was about 500 copies of target template per reaction, while for qRT-PCR the limit was only 10 copies per reaction (Harper et al., 2010).

### Genetic variation

In order to characterize and differentiate strains and pathotypes of *X. fastidiosa*, several molecular techniques have been used. In the late eighties and early nineties, restriction fragment length polymorphism (RFLP) and randomly amplified polymorphic DNA (RAPD) analyses were largely diffused to study strain differentiations of pathogenic bacteria(Denny et al., 1988; Hartung and Civerolo, 1989; Grajalmartin et al., 1993; Cave et al., 1994; Albibi et al., 1998; da Costa et al., 2000; Ferreira et al., 2000; Mehta et al., 2000; Lacava et al., 2001; Su et al., 2008). In a study on *X. fastidiosa*, RFLP were used to characterize 24 strains of this pathogen from 8 different hosts (Chen et al., 1992). In the same period RAPD analysis was also used, for the ease of this technique that doesn't require any *a priori* knowledge of the DNA sequence (Chen et al., 1995; Pooler and Hartung, 1995b, #708). In some cases RFLP and RAPD were also used together, for example to study sweet orange plants affected by CVC (Rosato et al., 1998). In the following years, RFLP and RAPD were often used in combination with other techniques, such as REP-PCR, ERIC-PCR and contour-clamped homogeneous electric field (CHEF), in order to improve sensitivity and reliability of the test (Chu et al., 1986; Versalovic et al., 1991; de Bruijn, 1992; Mehta et al., 2001; Qin et al., 2001) The results obtained with the combined techniques always showed greater resolution than using any single method alone (Hendson et al., 2001). Simple techniques such as RAPDs, usually are not suitable to distinguish between closely related strains of *X. Fastidiosa* and nowadays have become quite obsolete. Nevertheless, as no complex equipment or sequencing steps are needed, RAPD analysis could still be useful for routinely analysis, where this technique usually showed good resolution. As an example, a comparison between RAPD and 16S rDNA sequence analysis was made to study three groups of *X. fastidiosa* strains from citrus, grape and mulberry. Phylogenetic trees were obtained separately with the two techniques. Three distinct groups, from a total of 21 strains collected in Florida, Nebraska and Brasil, were detected, causing PD, CVC and MLS, respectively, using RAPD analysis, while sequence analysis of 16S rDNA only distinguished two groups, causing PD and CVC, while the MLS group was included in the PD group (Chen et al., 2002).

Even though more advanced techniques have been developed when high resolution is needed, PCR-RFLP was used till recent years to study *X. fastidiosa*. Four open reading frames (ORFs) related to the restriction modification type I system, ordinarily named R–M have been characterized. The study was carried out on 43 different strains isolated from citrus, coffee, grapevine, periwinkle, almond and plum trees and allowed to define haplotypes for all the loci. By analysis of molecular variance (AMOVA) two distinct groups were obtained, the first comprising populations from citrus and coffee plants, the second including populations isolated from grapevine, periwinkle, mulberry and plum. Moreover gene transfer was detected between coffee and citrus strains coming from southern Brazil, probably due to geographic proximity (Picchi et al., 2006).

A more recent technique to study genetic variability among strains exploits the so called short sequence repeats (SSRs) that are located within the prokaryotic genome (Kremer et al., 1999). Such short repetitive regions, due to the potentially variable number of tandem repeats (VNTR), can be highly polymorphic among different strains of bacteria and therefore represent a valuable tool for molecular studies.

A set of 9 SSR markers were developed within the genome of *X. fastidiosa* and used for genotyping studies (Table 4), comparing the results with the RAPD method (Della Coletta et al., 2001). The genetic diversity estimated using SSR markers was considerably higher than using RAPDs, as the former specifically target hypervariable regions. Similar differences in variability were obtained when SSRs and RAPDs were used to characterize populations of *X. fastidiosa* isolated from citrus in two different studies, one of which comprising a total of 360 bacterial strains (Della Coletta-Filho and Machado, 2002; Coletta and Machado, 2003). A second set of 34 SSR loci was identified in a genome-wide search, specific primers were designed (Table 4) and used to evaluate genetic diversity among 43 isolates of *X. fastidiosa* collected from grape, almond, citrus and oleander (Lin et al., 2005). Again, SSR markers proved to be powerful tools to distinguish genetically similar isolates, as the average level of polymorphism found among the 34 SSRs was 11.3 alleles per locus. Numerous studies on *X. fastidiosa* have been reported using SSR markers for genotyping strains infecting different plant species, such as grape (Lin et al., 2013), sweet orange (Coletta et al., 2014), almond (Lin et al., 2015), and coffee (Francisco et al., 2017), sometimes in combination with other techniques such as RFLP (Montero-Astua et al., 2007) (Table 4). In all cases, by the analysis of multiple SSR loci, it was possible to study temporal and spatial differences between closely related populations of *X. fastidiosa*, as well as population structure.

**Table 4:** List of SSR markers specific for *X. fastidiosa*.

**Marker**	**Forward primer**	**Reverse primer**	**Motif**	**Hosts**	**Citations WoS - G-Scholar**
SSR20^a^	ATGAAGAAGCCAGGATACAT	GCTACACGTGCAACAAC	(ATTGCTG)13	Citrus, coffee, grapevine, plum, Japanese Lantern, periwinkle	8–98
SSR21^a^	AACACGGATCAAGCTCATG	GGAACACGCAATAGTAAGA	(TGTTATC)21	–	
SSR26^a^	CTGTGATCGGTGAATTGA	TCAAGCACACTTCCTACG	(GTGTGTGA)37	–	
SSR28^a^	GCAACGCTGTTATCTCAAT	ATTACGCTTCTTATCGCTGT	(GTGTGCCT)11	–	
SSR30^a^	TACGCTGCACCTGTCTG	CTGTGAACTTCCATCAATCC	(TGATCCTG)15	–	
SSR36^a^	ATGTCACTCAGGTCAGG	CAGAACCACCGACTG	(TGTTGGGG)10	–	
SSR40^a^	ACCTTGACGACGGATG	TAGGAACTGCTGCTACTGAT	(GAAGGCGTA)27	–	
SSR32^a^	AGATGAACCTCGCCAC	GTACTCATCTGCGATGG	(CTGATGTG)9	–	
SSR34^a^	TGATAGAACTGTTTGACGCATTTG	TCGGGAAGTTTGGGGTGAC	(TTGGGTAG)22/(TTGGGTAA)35	–	
OSSR-2^b^	TTGCTTCACCATTAGCCTTATC	GGCCGTACAGGACCGATC	(ATG)9	Grape, citrus, almond, oleander	22–37
OSSR-9^b^	TAGGAATCGTGTTCAAACTG	TTACTATCGGCAGCAGAC	(TTTCCGT)13	–	
OSSR-12^b^	ACAGTCTGTGTCCGCAATTTG	CAGGCGCAGATAGCATTGATC	(AGAGGGTAT)9	–	
OSSR-14^b^	GGCGTAACGGAGGAAACG	ATGAACACCCGTACCTGG	(TGATCCATCCCTGTG)11	–	–
OSSR-16^b^	GCAAATAGCATGTACGAC	GTGTTGTGTATGTGTTGG	(CTGCTA)12	–	
OSSR-17^b^	AGTACAGCGAACAGGCATTG	AGCAACCAGGACGGGAAC	(TGCCTG)10	–	
OSSR-19^b^	GCTGTGAACTTCCATCAATCC	GCAAGTAGGGGTAAATGTGAC	(CAGGATCA)10	–	
OSSR-20^b^	ATCTGTGCGGCGGTTCTG	CACTTGCGGCGTAGATACTTC	(AGGATGCTA)20	–	
CSSR-4^b^	AACCCAATTCTTTTAATATGTG	TTGCAGCATTAGATATTTGAG	(TGCC)7	–	
CSSR-6^b^	CGCACTGTCATCCATTTAATC	GCTGCTTCATCTAGACGTG	(GCTGTA)7	–	
CSSR-7^b^	CACAGCGAACAGGCATTG	AGCAACCAAGACGGGAAC	(CTGTGC)14	–	
CSSR-10^b^	GCAACCACAAAGCCGCAG	AGCACCTCTTAGCATCACTGG	(CAATGA)10	–	
CSSR-12^b^	TAAGTCCATCACCGAGAAG	AAACGGATTTAGGAACACTC	(GAAGGCGTA)27	–	
CSSR-13^b^	CAATGTCACTCAGGTCAG	TTCTGGAATACATCAAATGC	(TGTTGGGG)10	–	
CSSR-16^b^	CGATCAACCCATTCACTG	GCTCCTATTTGCATGATATTG	(GTGGTGGCA)6	–	
CSSR-17^b^	AGAAGTATTCGCTACGCTACG	GGTGATGATTCAGTTGGTGTTG	(CTGATGTG)9	–	
CSSR-18^b^	GTGCTTCCAGAAGTTGTG	GACTGTTCTCTTCGTTCAG	(GCCAA)12	–	
CSSR-19^b^	TGCTGTGATTGGAGTTTTGC	TCAAACGAATCTGTCCATCAAG	(TGGTGAG)7	–	
CSSR-20^b^	GGTATCGCCTTTGGTTCTGG	GACAACCGACATCCTCATGG	(GTAGCA)8	–	
ASSR-9^b^	GGTTGTCGGGCTCATTCC	TTGTCACAGCATCACTATTCTC	(CAAGTAC)11	–	
ASSR-11^b^	AGAGGCAACGCAGGAACAG	GTGAGTTATATCGGTGCAGCAG	(ACGCATC)10	–	
ASSR-12^b^	TGCTCATTGTGGCGAAGG	CGCAACGTGCATTCATCG	(GATTCAG)14	–	
ASSR-14^b^	TTGACTCAAGGAATAAAAC	GAAAAGAGTGTCAATACG	(CTGCGTGC)11	–	
ASSR-16^b^	TTAATCAACAACGCTTATCC	TCGCAGTAGCCAGTATAC	(GCTCCGGTTCTA)26	–	
ASSR-19^b^	CGCCGACTGTCTATGTGAC	TTCCTAGCAATGGCAATGTTG	(ACAACG)10	–	
ASSR-20^b^	TTACTATCGGCAGCAGACG	TGAAGCAATGGTGGATTTAGG	(ACAGAAA)10	–	
GSSR-4^b^	GCGTTACTGGCGACAAAC	GCTCGTTCCTGACCTGTG	(ATCC)7	–	
GSSR-6^b^	TGTTCTCTTCGTTCAGCCAAGC	CGCAGCAGAGCAGCAGTG	(CTTGT)12	–	
GSSR-7^b^	ATCATGTCGTGTCGTTTC	CAATAAAGCACCGAATTAGC	(GGCAAC)24	–	
GSSR-12^b^	TTACGCTGATTGGCTGCATTG	GTCAAACACTGCCTATAGAGCG	(TATCTGT)20	–	
GSSR-14^b^	TTGATGTGCTTTTGCGGTAAG	GACAGGTCCTCTCATTGCG	(TCCCGTA)24	–	
GSSR-15^b^	CCGCAGAGTCCGTTGTAAC	AGCCGACGCACGGTATATC	(AGCCTGC)17	–	
GSSR-19^b^	GCCGATGCAGAACAAGAAC	TCAACTTCGCCACACCTG	(GAAAACAAG)19	–	
GSSR-20^b^	TGGATGGATAGATGATTCAGCC	CGATCAGTGGAGGATGTCTTG	(GAACCACTA)7	–	
COSS1^c^	GAAACAAGATGGCGGTTGC	CATTTAAACGGGCGGCATA	(ATTGCTG)15	Coffee, citrus	0–0
COSSR6^c^	TGCTGCGCGATAACCAAGT	CATCCAATCAGCCCTAACCT	(GTGATGCG)10	–	
CSSR45^c^	ACAGACATCACCGGCATTG	AATGTCGCTGCCAATCCAT	(CACACCGAGATGGAC)8	–	
COSSR4^c^	CAAGGTGACCGCTAGCCTAT	GCTGTCATTGGGTGATGC	(CAATACAC)13	–	
COSSR5^c^	ACACTGACACAACAGCCACCA	AATGGTGGGTGTGATGGTTTC	(CATACAGA)9	–	
COSSR3^c^	AAGTATTCGCTACGCTACGC	GTGTGTTATGTGTGCCATTCGT	(CTGATGTG)10	–	
CSSR42^c^	ATTACGCTGATTGGCTGCAT	GTTTCATTACGCGGAACAC	(TGTTATC)21	–	

a *Della Coletta-Filho et al. (2001)*.

b *Lin et al. (2005)*.

c *Francisco et al. (2017)*.

A less common but useful technique to characterize *X. fastidiosa* from a molecular point of view, especially considering the increasing amount of genomic sequences available, is represented by single nucleotide polymorphisms (SNPs) (Stoneking, 2001). A genome-wide search for SNPs and insertion/deletions (INDELs) using genome sequence information from four *X. fastidiosa* strains was performed (Doddapaneni et al., 2006). A total of 12,754 SNPs and 14,449 INDELs in the 1528 common genes and 20,779 SNPs and 10,075 INDELs in the 194 non-coding sequences were found. SNP markers were developed from 16 distinct genomic regions of 24 strains of *X. fastidiosa* isolated from coffee and citrus and positively used to discriminate among genetically close genotypes (Wickert et al., 2007). Combined use of SNPs and other techniques allowed to assess genetic diversity of *X. fastidiosa* strains from citrus and coffee plants (Montes-Borrego et al., 2015) and olive trees (Mang et al., 2016).

Another molecular approach that can be useful to infer phylogenetic relationships in bacteria is the characterization of the rDNA genetic locus. Such locus is of extreme importance for all organisms and moreover it is conserved enough to allow a universal classification of evolutionary relationships among species (Cedergren et al., 1988; LeblondBourget et al., 1996). 16S rDNA sequence analysis was often used to study inter- and intraspecific phylogenetic relationships in *X. fastidiosa*. The 16S rDNA sequences from 16 strains of *X. fastidiosa* isolated from 9 different hosts were amplified by PCR, cloned and sequenced. The results indicated that the strains could be divided into three groups, one including PD and MLS strains, the second including PLS, PPD, OLS, ELS, and perwinkle wilt strains and the third CVC and CLS strains (Chen et al., 2000a). A 20-bp oligonucleotide from the same sequence was also identified to be highly characteristic of *X. fastidiosa* and different from other bacteria, including the closely related *Xanthomonas* genus. Therefore, 16S rDNA was proposed as a signature character for the identification of *X. fastidiosa* (Chen et al., 2000b). In all, 16S rDNA sequence analysis can be considered a fast and reliable method for the identification of bacteria at genus or species level. Nevertheless, when genetic distances decrease under the species level, the sequence differences found in the 16S rDNA aren't always enough to distinguish between closely related strains. A good way to partially overcome this problemis to analyze also the 16S-23S ITS.This intergenic region presents a higher variation in length and sequence than 16S rDNA and therefore can be used to increase the sensitivity of the analysis (Garcia-Martinez et al., 1999; Jeng et al., 2001). In *X. fastidiosa*, combined sequence analysis of 16S-23S ITS and 16S rDNA was performed in strains from grape, citrus, coffee, plum, and pear (Mehta and Rosato, 2001). The level of similarity was indeed higher for 16S rDNA (97.1–100%) than in 16S-23S ITS (79.8–100%). Phylogenetic relationships based on 16S-23S ITS sequence analysis among strains of *X. fastidiosa* isolated from a number of different hosts have been studied (Huang and Sherald, 2004; Martinati et al., 2005). Randall et al. (2009) have analyzed several strains of *X. fastidiosa*, collected from New Mexico, California and Arizona and infecting *Chitalpa tashkentensis*, a common ornamental landscape plant used throughout the southwestern USA. By sequence analysis of 16S rDNA and 16S-23S ITS, a differentiation of chitalpa strains from all the known *X. fastidiosa* subspecies was highlighted and therefore a new subspecies (subsp. *tashke*) was proposed. The differentiation of chitalpa strains from other known strains was further analyzed by several approaches (such as analysis of *gyrB*, SSRs and the virulence-associated protein VapD) that sometimes gave ambiguous results, such as for example the VapD analysis, showing that the chitalpa isolates from New Mexico were more similar to the CVC strain than to the Arizona isolates. Since the first report, the newly proposed subsp. *tashke* has not been reported anymore and remains poorly characterized. Moreover, it must be noted that the distinction between subspecies is not always clear (see below), depending on the method used for the characterization and the fast evolution of bacterial populations.

*X. fastidiosa* was the first plant bacterium to have its complete genome sequenced (Simpson et al., 2000). Since then, a lot of efforts have been made to elucidate the complete genome sequence of several *X. fastidiosa* strains infecting different plant hosts, such as grape (Van Sluys et al., 2003), almond (Chen et al., 2010), mulberry (Guan et al., 2014b), sycamore (Guan et al., 2014a), pear (Su et al., 2014), coffee (Giampetruzzi et al., 2015b), and olive (Giampetruzzi et al., 2015a). This huge amount of data can be used for extensive *in silico* analysis in order to identify similarities and differences among strains at a whole genome scale. Such approach allowed the researchers to study *X. fastidiosa* strains with unprecedented resolution. By whole genome approach, the complete set of unique genes present in each strain can be characterized, together with those that are common to all strains (Bhattacharyya et al., 2002; Barbosa et al., 2015). Sets of genes involved in important functions can be compared and studied (da Silva et al., 2007; Barbosa et al., 2015), SNPs can be identified and PCR primers can be designed for the identification of specific strains (Doddapaneni et al., 2006; Marcelletti and Scortichini, 2016b) and detailed phylogenetic analysis can be performed. As an example, by genome-wide comparison of 21 *X. fastidiosa* strains, Marcelletti and Scortichini (2016a) constructed a phylogenetic tree analyzing 820,088 nucleotides, ~30% of the entire *X. fastidiosa* genome. According to their results, three different, clearly defined subspecies of *X: fastidiosa* can be identified, while the two subsp. *sandyi* and *morus* are actually members of the subsp. *fastidiosa* (Marcelletti and Scortichini, 2016a). Although extremely powerful, the whole genome analysis is still a time-consuming and relatively expensive technique that cannot be considered an applicable method for a fast identification of pathogens, especially in field applications. Instead, it could be very useful to perform a whole genome study when a new site of infection is found, especially in those Countries where *X. fastidiosa* was never reported before, in order to efficiently classify the strain and its origin (Marcelletti and Scortichini, 2016b).

A relatively new method for bacteria identification is multilocus sequence typing (MLST). It was developed at the end of the nineties and the characterization of different strains is based on nucleotide sequence differences in a small number of housekeeping genes, typically seven (Maiden et al., 1998). Briefly, in MLST each allele of a given gene is assigned a number, so different strains of bacteria can be characterized by a series of numbers, representing one allele for each locus analyzed. The combination of the allele numbers at each locus determine the so called sequence type (ST) for each analyzed strain. The advantages of MLST are high resolution and reproducibility, fast analysis and easy interpretation of data that usually are made available in a public database (http://pubmlst.org/xfastidiosa/). Moreover MLST datasets can be used to estimate the relative contributions of recombination and point mutations in the formation of new alleles within a closely related group of strains that is defined as clonal complex (Enright and Spratt, 1998; Feil et al., 2000, 2001, 2004). MLST was applied to 25 strains of *X. fastidiosa* from five different host plants: grape, oleander, oak, almond and peach. An initial set of 10 sequences was used (Table 5) and all the bacterial strains were grouped into six clonal complexes corresponding to previously identified phylogenetic clades (Scally et al., 2005). The same approach was used to study the evolutionary relationships, geographic variation, and divergence times among 26 *X. fastidiosa* isolates from grape, oleander, almond oak, peach, plum and citrus (Schuenzel et al., 2005). MLST approach was compared to SSRs in a study on 26 CVC and 20 CLS strains (Almeida et al., 2008). Although both SSRs and MLST provided similar results, the authors concluded that SSRs are more useful to study *X. fastidiosa* at population level, while MLST may be more suitable for strain/subspecies studies. In the last years, MLST was largely employed in population studies of *X. fastidiosa*, for example to better understand the genetic diversity of subsp. *fastidiosa* and subsp. *sandyi* (Yuan et al., 2010) or to clarify the role of mutation and homologous recombination in bacterial evolution (Nunney et al., 2012). The introduction of two new taxa of *X. fastidiosa* into Central America was documented, together with their introgression into the native subspecies (Nunney et al., 2014b). Recently, MLST was used to better understand the epidemiology of leaf scorch disease in a urban environment, by the analysis of 101 samples isolated from 84 trees of 7 different species located in Washington D.C. (Harris and Balci, 2015). An important evolutionary issue studied by MLST is intersubspecific homologous recombination (IHR) and how it can affect *X. fastidiosa* host specialization and host shifts (Nunney et al., 2013, 2014a,c). As an example, by MLST analysis of 21 *X. fastidiosa* isolates from mulberry, it was determined that such strains represent a distinct group, different from all the others described in literature. Therefore a new subspecies was proposed (subsp. *morus*), originating by IHR from *X. fastidiosa* subsp. *fastidiosa* and *X. fastidiosa* subsp. *multiplex* (Nunney et al., 2014c). As mentioned above, in a study combining MLST and whole genome analysis, Marcelletti and Scortichini (2016a) proposed that the subsp. *morus* is included in the subsp. *fastidiosa*, thus contrasting with the results from Nunney et al. (2014c). It is therefore conceivable that, due to the continuous gene flow by IHR among different *X. fastidiosa* subspecies, in the future it will become more and more difficult to determine a clear distinction between subspecies. As a matter of fact, a new subspecies of *X. fastidiosa* was recently described in pears and grapevine in Taiwan (Su et al., 2012, 2013, 2016; Almeida and Nunney, 2015). However, additional genetic typing is needed to fully support the recognition of this new subspecies and to further study the isolates in Taiwan (Nunney et al., 2012; Retchless et al., 2014).

**Table 5:** Housekeeping genes and specific primers used for multilocus sequence typing (MLST) in *X. fastidiosa*.

**Gene**	**Position**	**Forward primer**	**Reverse primer**	**Gene function**
holC^a^	XF0136	GATTTCCAAACCGCGCTTTC	TCATGTGCAGGCCGCGTCTCT	DNA polymerase III holoenzyme, chi subunit
rfbD^a^	XF0257	TTTGGTGATTGAGCCGAGGGT	CCATAAACGGCCGCTTTC	dTDP-4-dehydrorhamnose-3, 5-epimerase
nuoL^a^	XF0316	CATTATTGCCGGATTGTTAGG	GCGGGAAACATTACCAAGC	NADH-ubiquinone oxidoreductase, NQO12 subunit
nuoN^a^	XF0318	GGGTTAAACATTGCCGATCT	CGGGTTCCAAAGGATTCCTAA	NADH-ubiquinone oxidoreductase, NQO14 subunit
gltT^a^	XF0656	TTGGGTGTGGGTACGTTGCTG	CGCTGCCTCGTAAACCGTTGT	Glutamate symport protein
cysG^a^	XF0832	GGCGGCGGTAAGGTTG	GCGTATGTCTGTGCGGTGTGC	cysG Siroheme synthase
petC^a^	XF0910	CTGCCATTCGTTGAAGTACCT	CGTCCTCCCAATAAGCCT	Ubiquinol cytochrome c oxidoreductase
pilU^a^	XF1632	CAATGAAGATTCACGGCAATA	ATAGTTAATGGCTCCGCTATG	Twitching motility protein
leuA^a^	XF18188	GGGCGTAGACATTATCGAGAC	GTATCGTTGTGGCGTACACTG	2-Isopropylmalate synthase
lacF^a^	XF2447	TTGCTGGTCCTGCGGTGTTG	CCTCGGGTCATCACATAAGGC	ABC transporter sugar permease
rfbD^b^	XF0257	TTTGGTGATTGAGCCGAGGGT^a^	TCCATAAACGGCGCCTTC	dTDP-4-dehydrorhamnose-3, 5-epimerase
cysG^b^	XF0832	GGCGGCGGTAAGGTTG^a^	GCATATGTCTGTGCGGTGTGC	cysG Siroheme synthase
holC^c^	XF0136	ATGGCACGCGCCGACTTCT	ATGTCGTGTTTGTTCATGTGCAGG	DNA polymerase III holoenzyme, chi subunit
nuoL^c^	XF0316	TAGCGACTTACGGTTACTGGGC	ACCACCGATCCACAACGCAT	NADH-ubiquinone oxidoreductase, NQO12 subunit
gltT^c^	XF0656	TCATGATCCAAATCACTCGCTT	ACTGGACGCTGCCTCGTAAACC	Glutamate symport protein
cysG^c^	XF0832	GCCGAAGCAGTGCTGGAAG	GCCATTTTCGATCAGTGCAAAAG	cysG Siroheme synthase
petC^c^	XF0910	GCTGCCATTCGTTGAAGTACCT	GCACGTCCTCCCAATAAGCCT	Ubiquinol cytochrome c oxidoreductase
leuA^c^	XF18188	GGTGCACGCCAAATCGAATG	GTATCGTTGTGGCGTACACTG	2-Isopropylmalate synthase
malF^c^	XF2447	TTGCTGGTCCTGCGGTGTTG	GACAGCAGAAGCACGTCCCAGAT	ABC transporter sugar permease
pilU^c^	XF1632	CCGTAATCACAACTCAACAGGACA	CTGCGAATCAGCATGGCGTA	Twitching motility protein
acvB^d^	PD_1902	ACAGTATCGCCGTCGAAGTGATGA	CATGCATACRGCGATGYTTCCGAT	Virulence protein: suggested to regulate pathogenicity
copB^d^	PD_0101	ATGAACACCCGTACCTGGTTCGTA	ATTTAGTCTCCACCATGAGCCGCA	Copper resistance protein B precursor
cvaCa^d^	PD_0215	TGCGTGAATTRACATTGACCG	CCTAGTCTGCGGCTTAAGCAGATT	Colicin V precursor
fimA^d^	PD_0062	CCCAGTGCGTCGTTATCGATTATTGGT	TTTYGYACTCTCAAGCATCGCATC	Fimbrial subunit precursor
gaa^d^	PD_0315	TGAGAGCTGCYGATGTTCCAATGA	ACAGCTTCTGGCAAGAACAAGCAC	Glutaryl-7-aminocephalosporanic acid acylase precursor
pglAa^d^	PD_1485	TAGTGCTGGCCTAACGATGTYGGT	CCGTATCAGCAACCACATGGAAGT	Polygalacturonase precursor
pilA^d^	PD_1924	ATCGCKCTGCCYATGTACCAAA	CAGCATTGATCGTRTTGCTGTRTG	Fimbrial protein
rpfF^d^	PD_0407	GCGCTCCATAGTTCGGAGTGATTT	ATGTCCGCTGTACATCCCATTCCT	Regulator of pathogenicity factors
xadAa^d^	PD_0731	TGGGAGGTCAAAGYACTGCCATCA	GCATTGGCAGCAACACTCGAATCA	Outer membrane afimbrial adhesin
gltT^e^	PD1516	TTTTTCAGGGGTGTCGCGC	TTCCAACGTTACTGGACGCT	Glutamate symport protein
cysG^e^	PD1840	CCAAACATAGAAGCACGCCG	CGTATGTCTGTGCGGTGTG	Siroheme synthase
leuA^e^	PD1047	GGCCAGTGCTGTGTTTTGTT	GGGCTACTTGCTGGAGGAAG	2-Isopropylmalate synthase
lacF^e^	PD1465	TTCTTTGGTGGGTTGGGTGT	CACACAGCATCAACGTCGTC	ABC transporter sugar permease

a *Schuenzel et al. (2005)*.

b *Almeida et al. (2008)*.

c *Yuan et al. (2010)*.

d *Parker et al. (2012)*.

e *Harris and Balci (2015)*.

Moreover, new variants of *X. fastidiosa* infecting coffee were isolated and characterized in South America (Jacques et al., 2016; Bergsma-Vlami et al., 2017). In any case, the genetic diversity of *X. fastidiosa* is probably broad and still poorly characterized as suggested by a very recent work, studying genetic diversity of subsp. *pauca* and *multiplex* in several locations and plant hosts in South America (Coletta-Filho et al., 2017). By MLST analysis, the authors could identify 7 new ST for subsp. *pauca* and 2 new ST for subsp. *multiplex*. Also in this work, IHR between the two subspecies was observed, suggesting that in *X. fastidiosa* gene flow can occur in short time, as the subsp. *multiplex* is thought to be introduced in South America from North America and it was first reported only in the seventies (French, 1978). Despite all the recent works suggest that MLST is a very effective technique that can be used to successfully study *X. fastidiosa* genetic diversity, especially at subspecies level, one possible limitation is due to the fact that the housekeeping genes used for the analysis are usually relatively conserved among strains, limiting the resolution power when studying closely related genotypes. One possible way to overcome this problem is by multilocus sequence analysis (MLSA) of genes influenced by environmental factors, termed environmentally mediated genes. Such genes are supposed to be subject to positive selection pressure and therefore to have a greater sequence variability than conserved housekeeping genes (Hoffmann and Willi, 2008). Multilocus sequence analysis of environmentally mediated genes (MLSA-E) was applied to 54 *X. fastidiosa* strains isolated from different species (Parker et al., 2012). Nine genes that resulted from previous studies to have a role in the infection process and to be environmentally mediated were selected, amplified by specific primers (Table 5) and sequenced. Phylogenetic results indicated that MLSA-E can increase differentiation of *X. fastidiosa* isolates when compared with MLST and identify novel variations within the same subspecies. In another work, MLST was combined with the analysis of 6 genes involved in different biochemical functions in order to increase the genetic information and therefore the resolution power of the experiment (Elbeaino et al., 2014). Such approach was used for characterizing *X. fastidiosa* isolated from olive trees affected by OQDS in Italy and the results showed that olive strain is distinct from the four previously defined species *fastidiosa, multiplex, sandyi* and *pauca*, showing the highest level of similarity with the latter. In conclusion, at present MLST is probably the best PCR-based approach to identify and classify *X. fastidiosa*, as testified by the wide use of this technique in analyzing the new strains identified in the recent outbreaks of this pathogen in Europe (Elbeaino et al., 2014; Marcelletti and Scortichini, 2016a; Denancé et al., 2017). The diffusion of MLST will be probably even wider in the next years as the drawbacks of the method, that are mainly due to costs and availability of an automated DNA sequencer, will be overcome very quickly by the fast evolution and cost reduction that this kind of technology is undergoing in the last years. Nevertheless, for the same reasons, it is conceivable that in the close future whole genome approaches will gradually become the classification method of choice, due to the superior level of sensitivity and amount of information that can be provided by such techniques.

## Conclusion

*X. fastidiosa* has recently emerged as a potential threat to European agriculture after its outbreak in southern Italy were it was found associated to OQDS (Saponari et al., 2013; Cariddi et al., 2014; Elbeaino et al., 2014; Martelli et al., 2016). Among the most dangerous features of this pathogen are: (i) the host range is very wide, as *X. fastidiosa* is capable to infect more than 300 plant species; (ii) the initial stages of infection are often symptomless, so there's a high risk of contaminating new areas by importation of diseased plants; (iii) bacteria can be irregularly distributed in host tissues making the pathogen difficult to be detected; (iv) many insects of the C*icadellidae* family are capable of transmitting *X. fastidiosa*, including some European species such as *Cicadella viridis* and *Philaenus spumarius* (Saponari et al., 2014). Therefore, it's become mandatory to strengthen the surveillance, looking for infected plant material, in order to limit the spread of this pathogen. Moreover, the geographic expansion of the olive epidemic area in Italy should encourage new studies to identify possible vectors and hosts of *X. fastidiosa* in this newly colonized environment. DNA-based molecular approaches have proven to be a valuable tool for detection and characterization of plant pathogens and in the last years they are gradually substituting the traditional serological and biochemical methods. Even though some limitations still exist, the fast development of new and more sensitive technologies, a concomitant reduction of costs and a greater ease of use, together with the increasing amount of *Xylella*'s genomic sequences available (Simpson et al., 2000; Bhattacharyya et al., 2002; Van Sluys et al., 2003; Chen et al., 2010; Barbosa et al., 2015; Giampetruzzi et al., 2015a) will allow a further diffusion of the DNA-based methods for studying the ecology, epidemiology and taxonomy of *X. fastidiosa*. New researches on topics such as bacteria distribution, identification and classification of new strains, host specificity and transmission vectors will be of primary importance in order to efficiently fight this fastidious pathogen.

## Author contributions

Both authors have made substantial, direct and intellectual contribution to the work, and approved it for publication.

### Conflict of interest statement

The authors declare that the research was conducted in the absence of any commercial or financial relationships that could be construed as a potential conflict of interest.
